# A Decade of Avian Influenza in Bangladesh: Where Are We Now?

**DOI:** 10.3390/tropicalmed4030119

**Published:** 2019-09-11

**Authors:** Nadia A. Rimi, Md. Zakiul Hassan, Sukanta Chowdhury, Mahmudur Rahman, Rebeca Sultana, Paritosh K. Biswas, Nitish C. Debnath, SK Shaheenur Islam, Allen G. Ross

**Affiliations:** 1 icddr,b, Dhaka 1212, Bangladesh; zhassan@icddrb.org (M.Z.H.); sukanta@icddrb.org (S.C.); rahman.mahmudur@icddrb.org (M.R.); rebeca@icddrb.org (R.S.); allen.ross@icddrb.org (A.G.R.); 2 Department of Microbiology and Veterinary Public Health, Chattogram Veterinary and Animal Sciences University, Chittagong 4225, Bangladesh; biswaspk2000@yahoo.com (P.K.B.); principalcgvc@gmail.com (N.C.D.); 3 Department of Livestock Services, Ministry of Fisheries and Livestock, Dhaka 1215, Bangladesh; s_islam73@live.com

**Keywords:** avian influenza, Bangladesh, biosecurity, H5N1, poultry, surveillance, vaccination

## Abstract

Highly pathogenic avian influenza (HPAI) has been a public health threat in Bangladesh since the first reported outbreak in poultry in 2007. The country has undertaken numerous efforts to detect, track, and combat avian influenza viruses (AIVs). The predominant genotype of the H5N1 viruses is clade 2.3.2.1a. The persistent changing of clades of the circulating H5N1 strains suggests probable mutations that might have been occurring over time. Surveillance has provided evidence that the virus has persistently prevailed in all sectors and caused discontinuous infections. The presence of AIV in live bird markets has been detected persistently. Weak biosecurity in the poultry sector is linked with resource limitation, low risk perception, and short-term sporadic interventions. Controlling avian influenza necessitates a concerted multi-sector ‘One Health’ approach that includes the government and key stakeholders.

## 1. Background

Bangladesh reported its first outbreak of highly pathogenic avian influenza (HPAI) in poultry in 2007 [1]. Since then, a total 556 outbreaks of HPAI H5N1 in poultry have been reported in 52 of the 64 districts until 2013, and the virus has now became enzootic in poultry [1,2]. The other subtypes isolated were H1N2, H1N3, H3N6, H4N2, H5N6, H10N7, and the predominant low pathogenic avian influenza (LPAI) virus H9N2 [3,4]. Unusual mortalities caused by H5N1 have been reported in commercial poultry [5], waterfowl [6], and in crows [7]. Evidence of past exposure to H5 virus in nomadic ducks has been reported [8]. A total of eight human cases attributed to the subtype have also been reported since 2008 [9]. Bangladesh reported three mild human cases of H9N2 [10]. An outbreak investigation during 2012–2013 showed that detectable avian influenza viruses (AIV) RNA was found in nasopharyngeal swabs of 4.5% and on arm swabs of 18.5% of 371 asymptomatic poultry workers [11].

The complex nature of the poultry production and marketing systems, limited veterinary capacity, and low level of commitment from the raisers to report mortality to the government favor the persistence of H5N1 [12,13]. Every introduction of AIV into humans poses a risk of coinfection and genetic reassortment with co-circulating human influenza viruses, which could lead to the emergence of a novel influenza viral strain with pandemic potential [14]. There are three prerequisites for the emergence of a new influenza pandemic: (i) the emergence of a novel virus to which humans are widely susceptible; (ii) the new virus is able to replicate and cause disease in humans; and (iii) the new virus is transmitted efficiently from human-to-human [15]. Although effective human–human transmission of HPAI virus is not evident, the high population density and close contact between humans and animals in Bangladesh poses a pandemic threat [16,17].

In order to combat AIV, the Government of Bangladesh (GoB) adopted the first national preparedness and response plan in 2006 [18]. Since then, there have been numerous efforts to detect, track, and combat AIV from several government and non-government organizations. However, it is yet to be understood how much has changed since the advent of AIV in Bangladesh. This review discusses the history of avian influenza over the past decade in Bangladesh and demonstrates where we are now.

## 2. Clades of HPAI H5N1 Detected in Bangladesh

Several studies explored the genetic characterization of the HPAI H5N1 virus circulating in Bangladesh. The circulating HPAI H5N1 viruses in Bangladesh clustered with gs/GD clade 2.2.2 from February 2007 until the end of 2010. At the beginning of 2011, new incursions of viruses of clades 2.3.2.1 and 2.3.4.2 were detected in chickens, quails, ducks, crows, and migratory birds [19,20,21]. According to a phylogenetic analysis of the isolates of 2012 and 2013, all the isolates exclusively belonged to clade 2.3.2.1 [21]. By the end of 2014, circulating Bangladeshi H5N1 viruses exclusively belonged to clade 2.3.2.1a [22,23]. A more recently determined status of circulating AIV in Bangladesh from a surveillance of live bird markets (LBMs) and waterfowl in wetland areas from February 2015 through February 2016 revealed that a new genotype of H5N1 viruses, clade 2.3.2.1a, had become predominant [24]. These newly emerged H5N1 viruses contained the hemagglutinin, neuraminidase, and matrix genes of circulating 2.3.2.1a Bangladeshi H5N1 viruses and five other genes of low pathogenic Eurasian-lineage AIV, some of which were closely related to the genes of the strains isolated from ducks and wild birds from northeastern Bangladesh [24].

## 3. Surveillance

### 3.1. Poultry Surveillance

Since HPAI represents an important threat to human health, it is essential to characterize the different strains of AIV that are circulating in poultry. As part of the influenza preparedness and response plan, the Department of Livestock Services (DLS), in collaboration with other partners and donor organizations, strengthened the existing passive surveillance system and initiated an active surveillance program to rapidly detect HPAI H5N1 outbreaks in both commercial and backyard poultry in 2008 (Table 1). Through active surveillance, DLS supported the monitoring of 306 high-risk sub-districts out of 487 in Bangladesh, with support from Sweden, the United States Agency for International Development (USAID), World Bank, and Food and Agriculture Organization (FAO) [25,26]. Community Animal Health Workers (CAHWs), additional veterinary surgeons (AVSs), and Upazila Livestock Officers (ULOs) were trained to collect data and report on morbidity and mortality in poultry using a short message service (SMS) gateway system (i.e., a method of sending and receiving messages between computers and mobile phones) at the end of each working day. A central surveillance team at the DLS reviewed the internet-based SMS outputs to monitor trends in disease, morbidity, and mortality in poultry. This real-time reporting using SMS identified and contained 550 HPAI H5N1 outbreaks, entailing the culling of a total of 3.46 million poultry, and destruction of 2.97 million eggs belonging to 822 farmers. The system facilitated the reduction of the outbreak response time from 4.8 days to 1.4 days and captured 86% of the outbreaks [25]. The initiative continued until 2013 [26].

To strengthen the government surveillance system, the icddr,b, with funding and technical support from the US Centers for Disease Control and Prevention (CDC), has also been performing an LBM-based sentinel surveillance for AIV in poultry since 2007, in collaboration with the DLS, which included specimen and data collection, diagnosis, training, and research on AIV (Table 1). The primary objective of the surveillance is to identify AIV strains that are circulating in the LBMs and domestic poultry within Bangladesh. Initially one sub-district of Netrokona district was selected for sampling and data collection from poultry, based on the presence of mixed populations of domestic and wild birds. The surveillance was expanded to other sites, including Dhaka, Gazipur, Rajshahi, Dinajpur, and Chittagong. The surveillance program is still ongoing, with consistent funding support from the CDC. From 2007–2018, the surveillance has reported year-round detection of AIV, including HPAI H5N1, in waterfowl, commercial chickens, backyard chickens, and pool environmental swabs [27].

In 2016, the animal and human health services of the GoB, in collaboration with FAO, developed a method called ‘sink surveillance’ to detect AIV using pooled environmental samples in the LBMs of Dhaka and Chittagong (Table 1). LBMs are identified as the pathogen sink area, i.e., common locations where HPAI and LPAI viruses accumulate from various sources (poultry farms and backyards) from different parts of the country. The sink surveillance aims to eliminate the need to find the pathogens at source farms or for farmers to report suspected outbreaks. The surveillance was later expanded to other cities in Bangladesh. A joint team of animal health and human health government officials visited 106 LBMs on a monthly basis to collect environmental specimens. From the 708 pooled environmental samples from 33 LBMs of Dhaka, the surveillance identified 87.9% of the LBMs positive for influenza A, 39.4% positive for H5, and 21.2% positive for H9 [30]. This surveillance is presently ongoing [31].

There have been some efforts to track AIV in wild birds as well. The US Geological Survey, in coordination with FAO and icddr,b, conducted a wild bird survey in 2011 [26]. During 2010–2012, icddr,b, in collaboration with EcoHealth Alliance, conducted a survey of wild birds and domestic ducks in freshwater wetlands in northern Bangladesh and coastal areas of the Bay of Bengals to assess the prevalence of AIV, quantify flight distances, and trace the migratory routes of influenza virus-infected waterfowl [32]. Findings of the survey suggest that both migratory wild birds and domestic ducks in Bangladesh can harbor and shed influenza A viruses and the migratory waterfowl routes connect Bangladesh with other regions in south and central Asia. Another study conducted during 2012–2015 assessed the prevalence of AIV and antibodies against the virus among wild and domestic birds. The study found a higher AIV antibody prevalence in domestic birds than in wild birds, suggesting that domestic birds may be an important reservoir of the virus in Bangladesh, potentially exceeding the role of wild birds [33].

### 3.2. Surveillance for Human Infection with AIVs

LBMs are the primary hub for poultry marketing across Bangladesh [17], and also serve as a place of human–bird interactions. Studies have identified LBMs as the reservoir of both LPAI and HPAI H5N1 and an important source of transmission [34,35]. Since Bangladeshi LBM workers are at risk of AIV infection due to the ongoing circulation of these viruses among poultry in markets and their occupational exposure to poultry, the icddr,b, in collaboration with the Institute of Epidemiology, Disease Control, and Research (IEDCR) and DLS, initiated an active influenza surveillance among LBM workers and their household members in 16 LBMs in Dhaka in 2012 (Table 1) [28,36]. These markets were selected because they served as sentinel sites for existing AIV surveillance in poultry, and hence served as a ‘One Health’ platform to monitor the circulation of AIV both in poultry and in market workers. The objectives of the LBM workers’ surveillance were to identify human cases of AIV infection, to detect circulating AIV, and to assess serological evidence of AIV infections. This surveillance reported an annual incidence of 24 AIV RNA detections per 1000 LBM workers. Approximately 2% (9/404) of workers at LBMs in Dhaka were found to have seroconverted to H5N1 [28]. Three of the eight H5N1 cases and one of the two H9 cases reported to the World Health Organization (WHO) were detected through this surveillance. However, all H5 and H9 cases identified had mild illness [36]. This poultry worker component of this surveillance has been discontinued since 2017 due to lack of funding.

In 2007, icddr,b, in collaboration with the IEDCR and supported by the US CDC, established a hospital-based influenza surveillance (HBIS) in 12 tertiary care hospitals across Bangladesh to identify individuals and clusters of people with life-threatening infections with influenza virus and to characterize the diversity of strains circulating in Bangladesh [29,37]. The surveillance is currently operational in nine sites―seven government and two private hospitals. One human H5N1 case has been detected through this surveillance. The platform of National Influenza Surveillance, Bangladesh (NISB) was initiated by IEDCR in 2010 [29,37]. The primary objective of this surveillance is to identify strains of the influenza virus circulating in Bangladesh. Patients who meet the case definition of influenza-like illness (ILI) and severe acute respiratory illness (SARI) were enrolled. Currently, NISB is being carried out in 10 sentinel sites, all of which are district hospitals, except Dhaka Medical College Hospital (DMCH). No H5 subtype was detected though this surveillance. From both HBIS and NISB, epidemiological data are shared to FluID and virological data are provided to FluNet through the National Influenza Center (NIC) of the Global Influenza Surveillance and Response System (GISRS). Monthly routine surveillance reports are generated and shared with the collaborating hospitals and institutes, US-CDC, and WHO.

## 4. Biosecurity

Biosecurity measures can play an important role in preventing AIV in poultry and thus reduce the risk of potential zoonotic transmission to humans [38,39]. FAO defines biosecurity as the “implementation of practices that create barriers in order to reduce the risk of the introduction and spread of disease agents”; biosecurity in poultry farming requires “the adoption of a set of attitudes and behaviors by people to reduce risk in all activities involving domestic, captive exotic, and wild birds and their products” [40]. According to FAO, three principle elements of biosecurity are segregation, cleaning, and disinfection [40].

### 4.1. Backyard Poultry Sector

**Figure 1:**
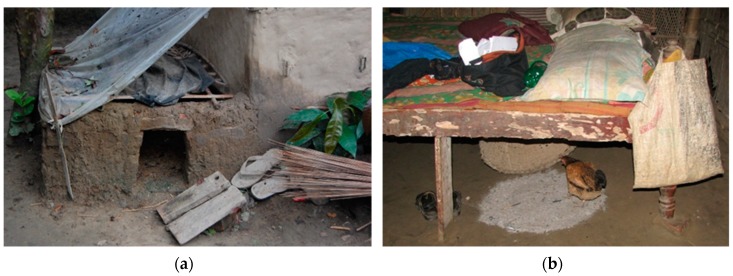
Backyard poultry farming: (**a**) backyard poultry shed; (**b**) backyard poultry kept under the bed.

In Bangladesh, 64% of the population live in rural villages [41], and approximately 71% of rural households raise backyard poultry (Figure 1) [42]. These backyard poultry raisers come into frequent close contact with poultry through their daily rearing practices, including putting poultry into sheds, feeding sick poultry by hand, and slaughtering sick poultry [43]. Given their limited resources and free scavenging method of raising, even the very basic biosecurity recommendations, such as controlling movement and traffic, separating sick poultry, maintaining regular cleaning, safe disposal and disinfection, are rarely feasible for backyard raisers [43,44,45,46], as observed in other similar settings [47]. Their close living arrangements with poultry put them at a heightened risk of zoonotic transmission. Several studies have identified a low awareness of AIV among the backyard poultry raisers, and biosecurity measures are seldom observed [42,48,49].

In the backyard sector, efforts have focused on raising awareness about AIV and measures to be followed to prevent zoonotic transmission [25,26]. The GoB, development partners, private sectors and non-governmental organizations (NGO), were involved in building awareness among communities with respect to biosecurity and HPAI (Table 2) [26,44,46,50,51,52,53,54]. 

Despite all these efforts, no significant improvement in biosecurity has been observed in this sector over time [66]. Two major underlying reasons for this low uptake of the standard recommendations were the low perception of the risk of AIV transmission to humans and concerns related to financial benefit or loss [43,44,48].

### 4.2. Commercial Poultry Sector

**Figure 2:**
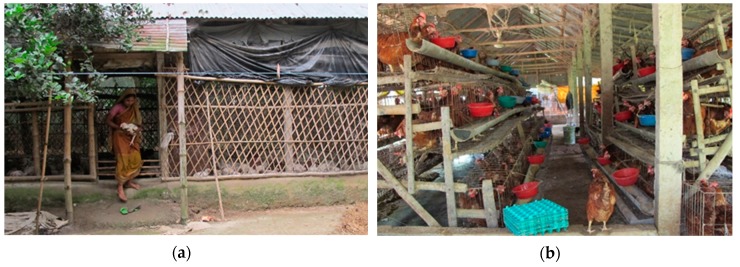
Commercial poultry farming: (**a**) a small commercial broiler farm; (**b**) a small commercial layer farm.

In Bangladesh, both large- and small-scale poultry producers have had to bear enormous losses associated with HPAI H5N1 outbreaks [46]. However, large-scale farms are better equipped to maintain biosecurity recommendations and withstand the financial loss due to sudden outbreaks compared to small-scale farmers. Small-scale commercial poultry farms (i.e., poultry population ≤ 2000) (Figure 2) account for 81% of the total commercial poultry farms [67]. Of the 549 confirmed outbreaks, 44% were among small commercial farms [5]. During 2007–2008, studies often characterized these farms with a low level of biosecurity in terms of the location of the farms, restricting the entry of wild birds and animals, fencing, use of footbaths, etc. [68,69], which match with the findings of another assessment conducted in 16 small commercial farms in 2011–2012 [57]. Environmental contamination was also reported through the use of untreated poultry feces as fertilizer in agricultural lands or as fish feed in waterbodies [57,70].

During 2007–2008, the GoB took a number of initiatives, including massive awareness-raising campaigns through mass media to promote the adoption of basic bio-security measures, and sub-contracting the private sectors, which worked at the grass-root level, to provide HPAI-related extension services in rural areas (Table 2). In 2010, the GoB recommended a set of biosecurity measures to reduce the introduction and spread of infectious diseases, including HPAI, into and from commercial poultry farms [71]. Other sectors, including development partners and NGOs, also joined the force [46,59,60]. There have been some individual efforts as well, for example, promoting community-based biosecurity measures by Upazila Livestock Officers (ULO), Kapasia, which reported to have markedly improved the HPAI outbreak situation in the sub-district [46].

Some improvements in farmers’ awareness and in some of the biosecurity conditions of the small commercial farms over the past decade have been reported, for example, maintaining the all-in-all-out system with the same broiler strain and age structure and some farm hygiene recommendations [72], as compared to findings from studies conducted during 2007–2008 [68,69]. However, the improvements are marginal and the overall biosecurity conditions of these small commercial farmers are still poor [72,73]. According to the World Bank report in 2013, weak poultry farm biosecurity and potential seasonal reinfection by the overflying and resting of HPAI-carrying migratory birds remained obstacles to successful control and eradication [26].

An anthropological exploration of 16 small commercial farms by icddr,b attempted to explore some underlying reasons for farmers’ low adherence to the standard measures during 2011–2012 [57]. The study showed that financial constraints and inconvenience were major constraints to practicing the recommended biosecurity measures. The study also showed that farmers’ practices and perception of biosecurity, transmission, and prevention of AIV were inconsistent with standard definitions, but were consistent with the recommendations and perceptions of the local vendors of chicks, feed, and medicines, indicating that these vendors heavily influenced farmers’ decisions [57]. A similar dependency on the local vendors was reported among the backyard poultry raisers in a previous study [74], indicating that this group is a key player for both sectors. These vendors, without any formal training, also prescribed antibiotics for poultry indiscriminately [57,74], contributing to the global concern for antibiotic resistance both for human and animal health [75]. 

### 4.3. Live Bird Market Sector

**Figure 3:**
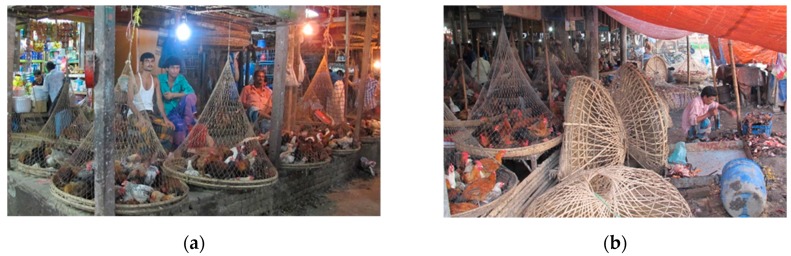
Live bird markets: (**a**) a live bird market in Dhaka; (**b**) slaughtering arrangements adjacent to poultry shops in a LBM.

LBMs represents 95% of the poultry meat and egg retail in Bangladesh [76], as refrigeration in the production, transport, and selling facilities is limited. As mentioned, these markets also act as a network ‘hub’ for poultry trading and potential reservoir of infection for poultry and poultry traders [17]. Bangladeshi LBMs were characterized as having a low level of biosecurity, lack of infrastructure required to maintain biosecurity, and low awareness of transmission, prevention, and risk perceptions associated with AIV (Figure 3) [56]. Waste from these LBMs also contributed to environmental contamination [56]. Among the eight reported cases of H5N1 in Bangladesh, three were LBM workers [77,78].

LBMs are probably the most targeted area for intervention by key stakeholders in order to prevent and control the spread of AIV. A number of intervention efforts have been made to improve biosecurity conditions over the past decade, including massive infrastructural renovation, the installation of short-term infrastructural solutions, market cleaning and disinfection, supplying personal protective equipment, promoting behavior change, and awareness campaigns (Table 2) [26,46,55,56,60,61,64,65]. Regardless of all the efforts, the biosecurity conditions in the LBMs remained low, despite the increased awareness [35,66,79], and the infection prevailed both in poultry and in the environment [80,81]. Evidence mentioned in the surveillance section above suggest that ongoing efforts for controlling HPAI did not have sufficient impact. Sayeed and colleagues identified housing chickens and ducks together in the stalls, birds kept on floors, and lack of adequate hygienic measures of the stall to be the crucial factors for spreading AIV in the LBMs of Chittagong [81]. Market closure or rest days and disinfection interventions were reported to be effective in disrupting the virus circulation in other settings [82], but could not be successfully implemented in Bangladeshi LBM [56].

## 5. Vaccination

Vaccination reduces the shedding of viruses. Unvaccinated infected chickens shed much higher concentrations of viruses than vaccinated infected chickens seven days post-vaccination [83,84]. Reduced quantities of virus shed into the environment, in turn, reduces human exposure and the likelihood of zoonotic transmission of the virus and pandemic influenza [85]. In parts of Asia, vaccination programs have been implemented and encouraged as part of an integrated control program for poultry [86]. The GoB and Breeder Association of Bangladesh introduced an avian influenza vaccine for the first time in commercial poultry farms of two districts in 2012. This vaccine was targeted for layers (raised for egg production), broilers (raised for meat production), and breeders, and applied to day-old chicks at hatcheries. The cost for a single dose of the vaccine was approximately BDT 5 (US$0.06) [87]. Since 2014, the Drug Administration Authority of the GoB has allowed restricted use of avian influenza vaccines for commercial poultry [88]. Since then, a vaccine against HPAI H5N1 has been available for use in commercial layers and breeder farms. However, it has been found that the virus can replicate and cause illnesses even in vaccinated birds. Ansari et al. showed that anti-H5 sero-positivity levels were similarly low in vaccinated and unvaccinated chickens, highlighting the need for a reevaluation of the currently available vaccine and the overall vaccination program [89].

## 6. Other Research

A number of epidemiological studies have been conducted to identify the risk factors associated with HPAI H5N1 in poultry in Bangladesh. Case-control studies conducted in Bangladesh have demonstrated several important risk factors—for backyard chickens: offering slaughter remnants of purchased chickens to backyard chickens, having a nearby water body, and having contact with pigeons [90]; for small commercial farms: the presence of dead crow at or near farms, exchanging egg-trays with market vendors, mortality seen in backyard chickens reared nearby [91], farms accessible to feral and wild animals and a footbath at the entry to the farm/shed [92]; and for layer farms: number of staff, frequency of veterinary visits, presence of village chickens roaming on the farm, and staff trading birds [93].

Studies that analyzed the temporal and spatial patterns of HPAI H5N1 outbreaks identified three significant circular clusters of hotspots located near large cities; the outbreaks were spatially clustered along the country’s main highways and principal poultry trading routes, with the central part of the country dominated by commercial production systems and the northwestern part primarily by backyard production systems [2,94,95]. Three significant risk factors associated with HPAI H5N1 virus outbreaks that were identified were the quadratic log-transformation of human population density, the log-transformation of the total commercial poultry population, and the number of roads per sub-district [2]. An ecological study identified migratory birds’ staging areas, river network, household density, literacy rate, poultry density, LBMs, and the highway network as ecological determinants significantly associated with the risk of HPAI-H5N1 outbreaks at sub-district level [96].

Efforts have been made to explore poultry workers’ and traders’ networks. A cross-sectional survey was conducted across 17 different districts of Bangladesh to assess poultry trading practices and networks, which could promote the spread of AIV, and their potential implications for disease control and surveillance [97]. The study showed that broiler chickens were generally sold in markets close to their production areas, whereas ducks and backyard chickens were moved over longer distances, and involved several intermediaries. The poultry trading network was highly connected, however, the removal of only nodes denoting 25 LBMs reduced the network’s connectedness, and the maximum size of output and input domains by more than 50%. Such knowledge of the network structure could be used to target control and surveillance interventions to a smaller number of areas, which could also be suitable for the optimum use of limited resources.

## 7. Avian Influenza Policy

During 2005–2006, the world was on high alert for AIV, and the United Nations (UN) agency encouraged every nation to develop its own avian influenza policies. With technical support from WHO and FAO, the GoB developed and adopted a National Avian Influenza and Human Pandemic Influenza Preparedness and Response Plan, covering the period from 2006–2008 [18] and then the period from 2009–2011 [98]. Both international guidelines and practices and local norms, experience, and evidence were considered while developing these avian influenza policies. A multi-sectoral approach was adopted to develop avian influenza policies in Bangladesh. The sectors that led the initiative from GoB included the Ministry of Health and Family Welfare, Ministry of Fisheries and Livestock, and the Ministry of Environment and Forests. Other stakeholders included international multilateral organizations, national and international NGOs, and trade associations, including breeders, feed millers, egg producers, and poultry farmers. Since then, the GoB has passed several ordinances during different outbreak situations, to be followed by different sectors.

UNICEF assisted GoB in the development of a risk communication strategy and USAID committed funds to finance different aspects of HPAI control. The Department of Mass Communication (DMC) under the Ministry of Information (MoI), in collaboration with DLS, implemented parts of the public awareness and information component. In 481 sub-districts, the DLS and MoHFW established joint rapid reaction teams to conduct the culling of exposed poultry, surveillance, and the administration of prophylaxis to exposed persons, and information sharing to minimize the threat to human health posed by the disease. The diagnostic capacity of the veterinary diagnostic laboratory system has also been strengthened [26].

Avian influenza has received more funding and attention than other zoonotic diseases, such as rabies and anthrax, which cause much higher mortality. Nevertheless, a trend of decreased attention towards AIV prevention and control efforts has been observed over recent years. After the most recent edition (2009–2011) of the AIV response plan was developed, attempts were made to update it but it is yet to be finalized. The major reasons behind AIV prevention and control interventions currently not being prioritized for policy implementation in Bangladesh could be the reduced number of human cases, low fatality among humans, and a perceived decreased trend in the number of outbreaks, despite under-reporting.

As part of a broader research on the Behavioural Adaptations in Live Poultry Trading and Farming Systems and Zoonoses Control in Bangladesh (BALZAC), funded as part of the Zoonoses and Emerging Livestock Systems (ZELS) project, a Chatham House roundtable was convened in Dhaka, Bangladesh in May 2016 [99]. Participants were convened from the government, international multilateral organizations, non-governmental organizations (NGOs), and trade associations in Bangladesh to discuss future policy options to prevent and control AIV and other poultry-related zoonotic diseases in Bangladesh. In the meeting, the policy options recommended were: (1) developing a broad overarching policy based on the One Health concept to cover a range of zoonotic diseases, with a subsidiary plan for each zoonotic disease; (2) using a bottom-up approach to develop policies considering local norms, experience, and scientific evidence; (3) developing sustainable policy through fostering a sense of ownership among those involved and exploring insurance options; and (4) reviewing and updating policy as necessary, including stocktaking and considering the effectiveness, cost-effectiveness, and acceptability of the policy. In order to consider and conceptualize a future policy environment suitable for developing and implementing such policies, the roundtable concluded that Bangladesh should take into account: (1) a multi-sectoral approach by establishing a One Health Secretariat in order to sustain the collaborative work between different sectors/organizations; (2) clearly defined leadership, roles, and responsibilities for each organization; and (3) the need for a common pool of resources and provision for transferring resources. Steps taken by partners to make progress since 2016 included the formation of the One Health Secretariat, Inter-ministerial Steering Committee on One Health, Technical Advisory Committee, and One Health Coordination Committee; the One Health Strategic Framework 2017–2021 being finalized; a revised National Avian and Pandemic Influenza Preparedness and Response Plan 2018–2022 under development; and a zoonotic disease prioritization workshop held to inform the development of an overall zoonoses policy.

## 8. Discussion

Over the last decade, Bangladesh has made a tremendous effort to combat avian influenza. However, it is evident that the viruses persistently prevailed in all sectors, caused sporadic infection, and continued to remain a public health problem. The apparently declining trend, based on officially confirmed reports since 2013 [1], does not carry any convincing evidence that the prevalence of the virus is decreasing over time, because an increasing yearly trend of its circulation in LBMs has been confirmed through different surveys and published reports. The persistent changing of clades of circulating H5N1 strains suggests probable mutations that might have occurred over time. Weak biosecurity in all the poultry sectors, linked with limited resources, low risk perception, short-term sporadic efforts, and decreasing attention toward AIV prevention and control among the stakeholders have all contributed negatively to the avian influenza situation in Bangladesh.

Avian influenza surveillances have provided evidence that HPAI H5N1 has become enzootic in Bangladesh. Despite the lack of actual disease reporting at the farm level, with the dominance of H9N2, different subtypes of AIV are being commonly identified at the LBMs [100,101]. The risk of transmission and reassortment of the viruses cannot be ruled out, considering the evidence of viable AIV found in the respiratory passages of the LBM workers [28,102] and the prolonged exposure [56]. To identify future reassortment in Bangladesh, monitoring for both HPAI and LPAI viruses of diverse subtypes will be crucial [100]. Although active surveillance can be expensive and time-consuming and may face difficulties surviving, the intensification of surveillance has been key to early detection and controlling and limiting the spread of HPAI viruses among poultry on national scale [54,103,104]. Active surveillance is also needed to track the likely chain of transmission and the genetic diversity of circulating strains. This will, in turn, contribute towards the standardization of sampling, testing, and reporting methods, bolstering full-genome sequencing efforts and encouraging the sharing of isolates with the scientific community [105,106]. Authorities might also consider exploring the potential value of enhancing surveillance for mild illness from HPAI H5N1 virus infection among humans during the typical AIV season in poultry. Capacity building in conducting whole genome sequencing is also important to predict whether the circulating virus strains have any potential to bind to human receptors.

Despite successive interventions to improve biosecurity conditions in commercial farms and LBMs by the government and the private sectors, risky behaviors remained widespread. It seems to be accepted among the stakeholders that ‘nothing can be done’ to improve the biosecurity conditions in the backyard poultry sector. On the other hand, there have been continuous efforts, although sporadic and disconcerted, to improve the conditions of the commercial poultry sector and LBMs, logically driven by concerns for larger scale financial investments.

The current biosecurity recommendations for commercial farms by the government includes different biosecurity measures for different types of commercial poultry sectors (e.g., grandparents, parents, layers, and broilers), however, the recommendations mostly include general measures for all farm sizes, which may not be practical for small farms [57,71]. To account for the socioeconomic realities of small-scale commercial farmers, biosecurity recommendations could be tailored [40]. Farmers’ dependency on the local vendors needs to be taken into account while developing any intervention for these small farmers [57,74]. Despite being an important contributor to this problem, the duck population has typically been ignored in terms of biosecurity interventions. Further research to develop and evaluate interventions that simultaneously improve duck raisers’ profitability and biosecurity should be considered.

LBMs remain a complex setting in terms of biosecurity improvements and to date no single intervention has been proven to be successful in the long term. This situation has instigated two different opinions. One opinion supports a gradual shifting of the poultry markets from selling live birds to marketing processed poultry meat, whereas the other supports retaining and improving the LBMs, considering the cultural preferences of the local people related to checking halal meat. The issue still remains unsettled and requires further behavioral studies and testing of small-scale interventions to identify approaches that can be acceptable, feasible, and support favorable conditions to maintain good biosecurity. At a minimum, interventions should prioritize creating a safer slaughtering environment in terms of disease transmission, and an improvement in sanitation and waste disposal facilities. Formative research could be helpful to explore if and how environmental controls (e.g., handwashing stands, improved ventilation flow), improved poultry handling and slaughtering techniques, and improved personal protective equipment (e.g., more accessible, cost-effective, and better tolerated) could help decrease the risk for AIV transmission at these markets.

Vaccination is another controversial issue. Some stakeholders favored vaccination in order to reduce the amount of circulating virus, which is important for an enzootic country like Bangladesh. However, others argued that vaccinated birds can still become infected and shed viruses with few or no clinical signs of infection [107], and the character of the virus might also change due to mutation. The complex infrastructures of the poultry industries and LBMs of some Asian countries made vaccination campaigns infeasible and HPAI enzootic in vaccinated poultry populations [108]. To prevent future AIV outbreaks in enzootic countries, vaccination campaigns need to be implemented along with biosecurity interventions. If the vaccination program is not properly managed with upgraded biosecurity, the prevention or control of AIV will not be possible [109]. For the high-risk LBM workers, a seasonal influenza vaccination can be considered to minimize the chances of a co-infection of seasonal and avian viruses and reduce the chances of re-assortment events, as seasonal viruses were also reported among the LBM workers [36]. Nevertheless, there is a strong need for impact evaluation and routine monitoring of vaccination. A Differentiating Infected from Vaccinated Animals (DIVA) program must be put into action to monitor the vaccine efficacy and natural infection.

## 9. Future Directions

Controlling AIV necessitates a concerted multi-sector One Health approach that includes human health, animal health, and environmental health to manage the health, social, and economic factors of the disease, since it affects poultry, humans, and the environment. In Bangladesh, there has been an increased acceptance of approaches or interventions that are effective against a combination of other zoonoses, such as Salmonella and Campylobacter, or other diseases that farmers are more concerned with, such as Newcastle and infectious bursal diseases [57], instead of AIV alone, among the donors and stakeholders. Responses to avian influenza has led to a longer-term trans-disciplinary One Health movement in many Asian and African countries [110,111], moving towards approaches that simultaneously address a variety of endemic zoonotic infections [110]. Multi-country networks, such as Mekong Basin Disease Surveillance (MBDS), have proven to be effective in regional cooperation and reporting, communicating, and containing disease outbreaks in isolated and economically marginalized border communities [110]. Failure to integrate and sustain One Health in national health policies in India has led to duplicative and weak response systems, failing to trigger investments and inadequate intersectoral action—a lesson for the developing countries with a significant burden of zoonoses, poverty, and a reliance on livestock [112]. Although Bangladesh has made significant progress in institutionalizing One Health, there are still some operationalization challenges which need to be mitigated in order to make it fully functional and sustainable. Multiple efforts are being undertaken by different stakeholders within the same sector in silos. Regular data sharing should be encouraged and maintained across government agencies and institutions, universities, research and multilateral organizations, and NGOs in order to secure the health benefits of all species.

## Figures and Tables

**Table 1:** Surveillance for poultry and human infections with avian influenza viruses.

Types of Surveillance	Species	Duration	Type of Samples Collected	Laboratory Tests Used	References
Poultry surveillance [icddr,b]	Waterfowl, commercial chickens, backyard chickens, market environment	2007–till date	Cloacal swabs, swabs from freshly laid feces, tracheal swabs, environmental pooled swabs	rRT-PCR for typing and subtyping of influenza A viruses	[27]
Poultry surveillance [DLS-FAO-ECTAD]	Waterfowl, commercial chickens, backyard chickens	2008–2013	Cloacal swabs, swabs from freshly laid feces, tracheal swabs	rRT-PCR for typing and subtyping of influenza A viruses	Personal communication, DLS
Sink surveillance [DLS-FAO-ECTAD]	Market environment	2016–till date	Environmental pooled swabs	rRT-PCR for typing and subtyping of influenza A viruses	Personal communication, DLS
Poultry worker’s surveillance [icddr,b]	Humans	2012–2017	Nasopharyngeal and throat swab (respiratory swabs), acute and convalescent blood specimens	Respiratory swabs: rRT-PCR for influenza A and B viruses and subtyping for influenza A Serum: haemagglutination inhibition (HI) and microneutralization (MN) assay	[28]
Hospital-based Influenza Surveillance (HBIS) [icddr,b]	Humans	2007–till date	Nasopharyngeal and throat swab	rRT-PCR for influenza A and B viruses and subtyping for influenza A	[29]
National Influenza Surveillance, Bangladesh (NISB) [IEDCR]	Humans	2010–till date	Nasopharyngeal and throat swab	rRT-PCR for influenza A and B viruses and subtyping for influenza A	[29]

**Table 2:** Initiatives to improve biosecurity in different poultry sectors.

Programs	Description	Results
Nationwide mass media campaigns *Duration:* 2007–2008 *Implemented by:* GoB, WHO, FAO, OIE, UNICEF, BRAC, CARE, USAID, AI.COMM, icddr,b, other NGOs *Targeted for:* All poultry sectors	Safe behaviors, 10-step recommendations (including basic hygiene messages, e.g., using masks, handwashing, and not touching sick poultry) were disseminated through radio, television, newspapers, public meetings, folk songs and plays, rickshaws and vans equipped with megaphones, posters, training manuals [46,50,51]	70% backyard and 90% commercial poultry farmers and 65% live bird handlers were aware of good biosecurity; 80% targeted journalists accepted good reporting practices; however, adoption of recommended practices remained poor in all sectors; 84% of HPAI outbreaks involving commercial farms indicated a disconnect between the KAP and practice as well as persisting weak biosecurity BPMC: some improvements in the structural biosecurity of the LBM and the farms under intervention was reported, however, operational biosecurity was poor for both the markets and the farms, and biosafety practices were almost absent [26,48,49,55,56,57]
Avian Influenza Preparedness and Response Project *Duration:* 2007–2012 *Implemented by:* DLS, Department of Mass Communications, Ministry of Fisheries and Livestock (MoFL), FAO *Targeted for:* All poultry sectors	Public awareness and risk communication campaigns conducted in 20 sub-districts in 20 districts using film shows, folk songs, school programs, distribution of leaflets, posters and banners; DLS trained poultry farmers, veterinarians, paraprofessionals, community health workers, media persons, news reporters, and students; piloted Biosecure Poultry Market Chains (BPMC) in 9 LBMs, 18 broiler and layer farms, among 324 poultry farmers, 180 LBM workers, 90 middlemen/transporters, and 1260 poultry chain stakeholders in 9 of the districts at highest risk of HPAI, to establish good biosecurity practices along the entire poultry value chain [26]	
Teacher training program for AI outbreak reporting *Duration:* 2009 *Implemented by:* FAO, DLS *Targeted for:* All poultry sectors	One-day workshops conducted in three selected sub-districts involving school and madrassa teachers on disease reporting and the risks and prevention of HPAI [52]	Not available
Behavior change pilot intervention *Duration:* 2009–2010 *Implemented by:* icddr,b *Targeted for:* Backyard poultry raisers	Context-appropriate behavior change recommendations piloted among the rural raisers in one community in each of the two districts [44]	Awareness increased but behavior remained unchanged; reasons for non-compliance: perceived absence of AIV in raisers’ flocks, low-risk of AIV, cost, inconvenience, personal discomfort, fear of being rebuked or ridiculed, and doubt about the necessity of the intervention [44]
Safe poultry slaughter pilot intervention *Duration:* 2014 *Implemented by:* icddr,b *Targeted for:* Rural communities	A safe poultry slaughtering method piloted in two rural communities in a district in order to reduce human exposure to airborne virus by performing poultry slaughtering in a closed container [53,58]	The recommendations were found to be acceptable and feasible for the villagers with minor modification [53]
Upazila-to-Community (U2C) *Duration:* 2017–till date *Implemented by:* DLS, FAO *Targeted for:* Backyard and commercial poultry sectors	Targeted to cover 496 sub-districts; avails veterinary services to rural communities to improve livestock production and disease control, increasing resilience to emerging disease events [54]	The program is still ongoing, no evaluation/result available
Program on farm biosecurity *Duration:* 2005–2006 *Implemented by:* GoB, DLS, BRAC and other NGOs *Targeted for:* Commercial poultry sector	Training on farm biosecurity (i.e., the prevention and control of AIV) provided along with gloves and disinfectants to 33 breeders/hatchery farm managers and 340 large commercial farms; 150,000 small-scale farmers trained across the country [46]	Not available
Stamping Out Pandemic and Avian Influenza (STOP AI) *Duration:* 2008–2010 *Implemented by:* USAID, FAO, city corporation, DLS *Targeted for:* Commercial poultry and LBM sectors	Different sectors were mobilized to improve biosecurity; biosecurity training implemented for veterinarians and livestock science graduates; 7 LBM training programs implemented in 5 divisions; cleaning and disinfection activities piloted in 2 LBMs; biosecurity improvement models (infrastructure improvements, e.g., farm boundary, footbath, biogas and compost plants) implemented in 12 commercial farms in a district and 2 LBMs in 2 districts; cleaning and disinfection activities implemented in 24 LBMs within and outside Dhaka through training, technical support, financial assistance for infrastructure renovations, renovation of the water supply, the addition of a biogas facility for proper waste disposal, and a slaughter house [54,59,60,61]	Awareness and precautionary practices increased; substantially fewer HPAI outbreaks were reported; no clusters of infection were found in the intervention farms/LBMs; the effect of the intervention on the incidence of disease was limited to a few months after completion—indicating the challenges of sustaining the progress; despite increased biosecurity, no significant reduction in virus circulation was found in the FAO-intervened markets compared to the non-intervened ones [60,62]
Community-engaged biosecurity (CEB) model *Duration:* 2016–2018 *Implemented by:* Bangladesh Agricultural University (BAU) *Targeted for:* Commercial poultry sector	From each of the two sub-districts, training of trainers (ToT) was provided to 50 lead farmers, who trained their fellow farmers; regular farm visits by community animal health workers were made to monitor compliance [63]	The program is still ongoing, no evaluation/result available
Biosecurity program in the LBMs *Duration:* 2007–2008 *Implemented by:* BRAC, IFC, SEDF *Targeted for:* LBM sector	A series of trainings and practical demonstrations on biosecurity and the use of personal protective equipment (PPE), along with gloves, masks, disinfectants, and small spray machines, were provided in retail and wholesale shops from 38 LBMs of Dhaka [46]	Not available
The LBM C4D initiative *Duration:* 2012–2013 *Implemented by:* UNICEF, GoB *Targeted for:* LBM sector	Intervention implemented in 16 LBMs to improve the knowledge and threat perception of AIV, as well as the bio-security practices of the poultry workers [56]	Despite an improved knowledge level, no significant change observed in biosecurity measures after the intervention; major barriers: lack of proper infrastructure to adopt the recommendations, concern of negative financial impact, lack of self-risk perception [56]
Piloting workstations for poultry workers *Duration:* 2008–2012 *Implemented by:* icddr,b *Targeted for:* LBM sector	Portable workstations (including a worktop and handwashing facility with soapy water) were designed and piloted in 13 shops in a LBM to reduce the risk of environmental contamination and improve handwashing practices [64,65]	The workstations were acceptable, functional, improved handwashing practices and the use of clean water; soapy water was effective in removing influenza viruses from poultry workers’ hands; however, handwashing decreased over time; major barriers: the difficulty to manage the increased cost for water and detergent by shops and the inability to frequently wash hands during busy hours [64,65]
Use of wooden shelters *Duration:* Not available *Implemented by:* BRAC *Targeted for:* Backyard poultry sector	Moveable wooden poultry shelters were developed and promoted to help the smallholder farmers to maintain bio-security measures at low costs [46]	Not available
